# Mesenchymal Stem Cells and Cardiovascular Disease: A Bench to Bedside Roadmap

**DOI:** 10.1155/2012/175979

**Published:** 2012-01-22

**Authors:** Manuel Mazo, Miriam Araña, Beatriz Pelacho, Felipe Prosper

**Affiliations:** Department of Hematology and Cell Therapy, Clínica Universidad de Navarra, Foundation for Applied Medical Research, University of Navarra, Avenida Pío XII 36, Pamplona, 31008 Navarra, Spain

## Abstract

In recent years, the incredible boost in stem cell research has kindled the expectations of both patients and physicians. Mesenchymal progenitors, owing to their availability, ease of manipulation, and therapeutic potential, have become one of the most attractive options for the treatment of a wide range of diseases, from cartilage defects to cardiac disorders. Moreover, their immunomodulatory capacity has opened up their allogenic use, consequently broadening the possibilities for their application. In this review, we will focus on their use in the therapy of myocardial infarction, looking at their characteristics, *in vitro* and *in vivo* mechanisms of action, as well as clinical trials.

## 1. Introduction

Although traditionally regarded as a health concern related particularly to the industrialized world, cardiovascular diseases are now the first cause of death worldwide [1], with myocardial infarction (MI) resulting in 12.8% of deaths. Aside from changes in ways of life associated with economic and social development, one of the main reasons is the fact that MI is an evolving disease. After the ischemic event, anaerobic conditions rapidly induce massive cell death, not only involving cardiomyocytes (CMs), but also vascular cells. Although the organism tries to exert a compensatory activity (reviewed in [2]) during the first stages of the disease and may even manage to partially restore functionality, the resulting scar is never repopulated, relentlessly leading the patient towards the setting of heart failure. Thus, though not conventionally regarded as such, cardiac disease is a degenerative affection in which lack of sufficient contractile and vascular cells leads to a decompensated neurohormonal microenvironment [3], which further impairs both organ function and cell survival.

Although the existence of stem cells has been a well-known fact for nearly half a century [4], it is in the last 15 years that the field has experienced a major boost. Their capacity for differentiation has made stem cells outstanding candidates for the treatment of degenerative diseases, substituting for cells lost during the course of the disorder. Consequently, cardiac diseases and MI have been the object of intense research [5]. Among the cell types studied, mesenchymal stem cells (MSCs) are strong candidates for success in the MI setting. In the following pages, we will discuss their capacities as well as pre- and clinical investigations in which these cells have been employed.

## 2. Origin, Types, and Characteristics

The studies by Friedenstein and colleagues are regarded as one of the first reports on MSC [4]. In these, the clonogenic potential of a population of bone marrow- (BM-) derived stromal cells, described as colony-forming unit fibroblasts, was examined. BM is indeed one of the best-known sources of progenitor cells, MSC being among them [6]. Although this is not entirely understood, BM-MSC are thought to act as supporters and nurturers of other cells within the marrow [7–9], possibly in a location close to blood vessels [10]. However, there is a relatively small population (0.01%–0.0001% of nucleated cells in human BM [11]), so MSC can be easily purified by plastic adherence and expanded after BM extraction. Similarly, but adding simple mechanical and enzymatic processing, a mixed cell population (called stromal vascular fraction, SVF) can be isolated from adipose depots, which, after *in vitro* culture and homogenization, gives rise to the mesenchymal progenitors from this tissue, also termed adipose-derived stem cells (ADSCs) [12]. Adipose tissue is regarded as a much richer source of progenitors, harboring 100 to 500 times the numbers seen in BM [13]. However, despite similarities in phenotype, differentiation, or growth kinetics, there are certain differences at a functional, genomic, and proteomic level [9, 14], suggesting a degree of higher commitment of BM-MSC to chondrogenic and osteogenic lineages than ADSC [15].

Adipose tissue and BM are the most widely researched sources of mesenchymal progenitors because they are easy to harvest, and owing to the relative abundance of progenitors and the lack of ethical concerns. Nevertheless, MSCs have been ubiquitously found in a variety of locations, as umbilical cord blood [16], dental pulp [17], menstrual blood [18], or heart [19], among others (reviewed in [20]). This wide variety of origins, methodologies, and acronyms prompted standardization in 2005 by the International Society for Cellular Therapy, which set the minimum requirements for MSC definition (Table 1). First, MSC must be plastic-adherent when maintained in standard culture conditions. Second, MSC must express CD105, CD73, and CD90, and lack expression of CD45, CD34, CD14 or CD11b, CD79a, or CD19 and HLA-DR surface molecules. Third, MSC must differentiate to osteoblasts, adipocytes, and chondroblasts *in vitro* [21]. Still, caution must be taken as some reports fail to meet these criteria, and MSC is often employed for “marrow stromal cell,” “mesenchymal stromal cell” or “marrow stem cell.” Accordingly, a clarification was published in which MSC was defined as “multipotent mesenchymal stromal Cells” [22], adding the supportive property to the required characteristics [23].

## 3. What Do MSCs Have to Offer to Cardiac Regeneration?

**Figure 1:**
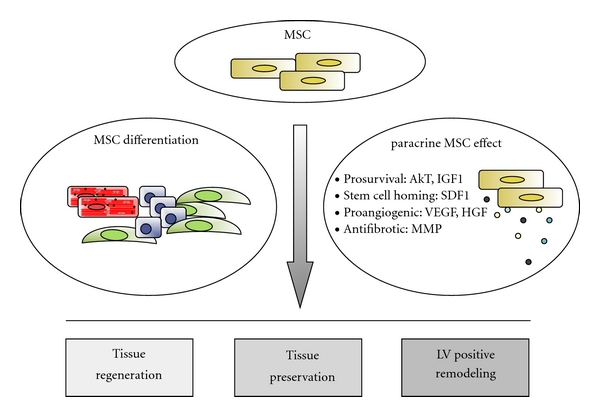
Main MSC actions on injured myocardium. Mesenchymal progenitors transplanted onto the ischemic myocardium are able to secrete a plethora of therapeutic molecules (paracrine activity) and even to differentiate towards (cardio-) vascular lineages, encouraging the healing of the damaged tissue, avoiding its transition to a scarred muscle, and regenerating the heart tissue mainly at the vascular level. Abbreviations: IGF-1: insulin-like growth factor-1; SDF-1: stromal derived factor-1; VEGF: vascular endothelial growth factor; HGF: hepatocyte growth factor; MMP: matrix metalloproteinase; LV: left ventricle.

When considering the goal of cardiac tissue regeneration, the desired objective must encompass three objectives: (i) the production of a replacement myocardial mass, (ii) the formation of a functional vascular network to sustain it, and (iii) the returning of the impaired ventricle to its proper geometry. Cell therapy may theoretically affect those processes in two ways: either by direct differentiation of transplanted cells towards the desired lineages or by their production of molecules with therapeutic potential (Figure 1).

BM-MSC have shown their *in vitro* capacity to give rise to endothelial cells (ECs) [24, 25] and smooth muscle cells (SMCs) [24]. Cardiomyocyte differentiation has proved more problematic, as either demethylating agents have been employed [26], or it has been inefficient and incomplete [27, 28]. In contrast, the cardiac potential of ADSC is better documented *in vitro*, showing their capacity to give rise to CM, either by the use of DMSO [29] or CM extracts [30]. In addition, ADSC seems to harbor a progenitor subset characterized by the expression of Nkx2.5 and Mcl2v [31] and whose differentiation relies on the autocrine/paracrine activity of vascular endothelial growth factor (VEGF) [32]. SMC [33] and EC [34] have been obtained from adipose cells, yet a cautionary note must be struck, as some of these studies either rely on subpopulations of freshly isolated cells or culture them in differentiation-promoting medium before purifying the mesenchymal population [35, 36]. Finally, other mesenchymal progenitors have also been differentiated to CM or CM-like cells, such as menstrual blood-derived MSC [18] or umbilical cord blood MSC [37].

However, although it is extremely interesting, this differentiation potential must cope with two opposing factors. First, patients receiving stem cell therapy are severely diseased and usually elderly, two factors that have an outstanding impact on stem cell function. For instance, a decrease in the numbers and functionality of circulating endothelial progenitors is directly related to cardiovascular risks and smoking [38, 39] and age has also been shown to impair the angiogenic capacity of both ADSC [40] and BM-MSC [41]. Second, the small percentage of engrafted cells (see [42] for a review) coupled to the huge catastrophe caused by an MI (the loss in some cases of over 1 billion CM [43]) and the low rate of differentiation achieved even under *in vitro* controlled conditions makes the adding of such small number of cells a therapeutically inefficient approach.

Nevertheless, secretion of beneficial molecules has been demonstrated to be able to exert a positive effect, even when a few engrafted cells are left [44]. These molecules can induce a benefit either by increasing tissue perfusion, decreasing collagen deposition and fibrosis, enhancing host-cell survival, or attracting/regulating endogenous progenitors. Thus, Chen and coworkers compared the expression profile of BM-MSC and dermal fibroblasts [45], showing that mesenchymal progenitors secreted a higher amount of several molecules, including the potent proangiogenic cytokine VEGF or the chemotactic stromal derived factor-1 (SDF-1). Conditioned medium from BM-MSC induced the recruitment of EC and macrophages, and improved wound healing. Moreover, it has recently been shown that serum-deprived BM-MSC acquire EC features and increase the release of VEGF or hepatocyte growth factor (HGF), another potent angiogenic molecule [46], both of which have been reported to be secreted by ADSC [32, 47, 48]. Moreover, Dr. March's group demonstrated that ADSCs have a pericytic nature and are able to form and stabilize functional vascular networks when mixed with endothelial progenitors [49]. Also, BM-MSC show a potent antifibrotic action, as their conditioned medium decreases cardiac fibroblast proliferation and expression of collagen types I and III [50, 51] and increases secretion of antifibrotic molecules such as matrix metalloproteinases (MMPs) 2, 9, and 14 [52]. These cells express five types of MMP (2, 13 and membrane type-MMP 1, 2, and 3) and are able to cross through type I collagen membranes [53], which theoretically would allow their trafficking across the infarction-derived scar. Likewise, ADSCs produce transforming growth factor- (TGF-) *β*1 [54], a potent regulator of fibrosis. Taken as a whole, these examples demonstrate that mesenchymal progenitors are potent paracrine mediators with a considerable capacity to impact infarct evolution.

One last noteworthy competence is the ability of BM-MSC and ADSC to modulate the immune response. Marrow-derived mesenchymal progenitors inhibit the proliferation of activated T cells and the formation of cytotoxic T cells [55], inducing an anti-inflammatory phenotype, which would allow their allogenic use and significantly broaden the scope of their applicability. However, Huang et al. reported that differentiation reduced their capacity of immunological escape [56], related to an increase of immunostimulatory molecules MHC-Ia and II and a decrease in the immunosuppressive MHCIb. Along similar lines, McIntosh and coworkers reported that ADCS beyond passage one (and thus devoid of contaminating differentiated cells [57]) failed to elicit a response from allogenic T cells [58], but this attribute may be diminished under inflammatory stimuli, as shown *in vitro* [59].

Finally, since the onset of induced pluripotent stem cells (iPSCs) [60], mesenchymal cells have been investigated [61, 62] due to their relatively easy harvest and higher potency than other cell types (e.g., dermal fibroblasts), which show an increased efficiency, even in the absence of the oncogene c-Myc. Their supportive capacities have also made them good candidates to replace mouse cells as feeders [63, 64].

## 4. MSC in Animal Models of MI

However, in spite of all the positive characteristics of mesenchymal progenitors already depicted, their *in vivo* testing in animal models of the disease is compulsory. In this regard, three different settings can be found. First, the acute setting, in which cells are transplanted within hours of the MI. Here, the inflammatory microenvironment and the necrotic/apoptotic signals released from resident cells [65, 66] are the main opposing forces to the therapeutic activity of cells. Nevertheless, homing signals [67] and an antifibrotic milieu [68] may have a positive influence. Also, from a practical point of view, dealing with acute models offers the advantage of subjecting animals to only one surgery, as at the time of the MI (or minutes after it), the cells are applied, thus decreasing mortality and invasiveness. As a consequence, the majority of published reports use acute models [37, 69–84]. Most studies (with the exception of the two by van der Bogt and colleagues [74, 77]) have consistently demonstrated that the treatment induces a significant benefit for cardiac function, mainly through paracrine mechanisms that induce an increase in tissue perfusion and a decrease in the size of the scar and collagen content.

Similar results have been obtained in a second setting, the chronic one. Here, the repair processes that take place after ischemia have been completed, the scar has matured, and although a new network of blood vessels has been created, this is disorganized and inadequate [85, 86]. These facts impose a great burden upon cell survival. However, it must be taken into account that the generation of homogeneous populations as BM-MSC or ADSC needs weeks of *in vitro* culture, thus, unless used in the allogeneic setting, there is no possibility of the bedside translation of the use of mesenchymal progenitors in the acute setting. In spite of this difficulty, fewer reports deal with this issue [87–90]. Compared to results in the acute setting, mesenchymal cell therapy of chronically infarcted hearts has a positive effect upon organ contractility and histology.

As a third and intermediate position, the so-called subacute model represents a situation where angiogenic processes are still on course, either through endothelial progenitors [91] or macrophages [92], and the receding of inflammation plus the increase in fibrotic processes are also on course. As with chronic models, there are few reports in this setting [17, 18, 93, 94], but again the benefit and mechanisms appear to be consistent.

Nevertheless, analyzing in more depth the studies mentioned above, it is possible to find a fair amount of information on how mesenchymal progenitors behave when injected into the diseased heart has been gathered. Chen et al. showed that transplantation of BM-MSC into chronically infarcted rabbit hearts induced an increase in the concentration of SDF-1 that elicited the chemotaxis of host-derived BM progenitors (CD34^+^, CD117^+^, STRO1^+^) and was related to a functional benefit, a decrease in infarct size and improvement in tissue vascularization [89]. Li and coworkers demonstrated that the functional enhancement was accompanied by the augmented expression of the prosurvival gene Akt [95] whereas Mias and colleagues showed that the benefit upon contractility and remodeling *in vivo* was accompanied *in vitro* by a plethora of antifibrotic actions [52]. In a sheep model of MI, the group of Dr. Spinale monitored the evolution of MMP and their inhibitors, demonstrating a relationship with the number of transplanted cells [75]. Resembling their *in vitro* behavior, several publications have demonstrated the association between proangiogenic activity *in vitro* and secretion (either direct or host-derived) of angiogenic cytokines as VEGF, HGF, or insulin-like growth factor-1 (IGF-1), among others [17, 84, 93, 96, 97]. Whether these capacities are related to the claimed pericytic nature of these cells [10, 48, 49] remains to be resolved.

Immune modulation (reviewed in [98]) in theory provides the means for the allogenic use of MSCs and as an off-the-shelf product (expanded prior to the onset of the ischemia and applicable on demand). Two reports have compared the effects of allogenic versus syngenic injection of BM-MSC in rat model of MI, with conflicting results. Imanishi et al. [78] demonstrated that both autologous and allogeneic cells improved cardiac function 4 weeks after transplantation, remained in the damaged tissues, and did not stimulate rejection. Huang and coworkers conversely [56] followed animals for up to 6 months. Syngenic cells stimulated cardiac recovery, but the effect of the allogenic treatment was transitory (significant 3 months after injection but not at 6) and BM-MSC disappeared earlier than their syngenic counterpart. However, this difference can be attributed to methodological discrepancies regarding time of transplantation (acute versus chronic resp.) or followup (1 versus 6 months). Equivalent and importantly, results from clinically relevant large animal models of MI in which allogenic cells have been employed have revealed either positive [99, 100] or no functional outcome [79]. In contrast, when autologous ADSC or BM-MSC are used [72, 83, 101, 102], reports have shown a robust and consistent functional recovery after cell transplantation. Thus, strict considerations about building up animal models must be taken into account.

## 5. Problems, Solutions

Despite all the optimism, stem cell therapy shows certain caveats that are amenable to improvement, namely, lack of substantial engraftment and cell persistence, high levels of death, and low *in vivo* differentiation capacity. Some approaches to try to remedy these problems have included the use of genetic manipulation and *in vitro* pretreatment of cells or biomaterials. In this sense, the CXCR4/SDF-1 axis has been greatly exploited. Ma et al. investigated the peak of cardiac SDF-1 expression [103] in rat MI, finding that injected cells at that time point (1 day postinfarction) increased cell engraftment and tissue angiogenesis. Cheng and coworkers transplanted BM-MSC engineered to overexpress the receptor CXCR4, strengthening cell homing to the injured tissue after tail vein injection [104]. The same group combined BM-MSC peripheral injection with administration of granulocyte colony-stimulating factor, which *in vitro* increased CXCR4 expression. However, although engraftment was increased, no effect of cardiac function was found [105]. Huang and associates demonstrated that overexpression of the chemokine receptor CCR1 but not CXCR2 was associated with improved survival and grafting in a mouse model of MI, which also restored functionality [106].

Cell survival in the infarcted myocardium is jeopardized by hypoxia, inflammation, or oxidative stress. Liu et al. engineered BM-MSC to overexpress angiogenin [107], which improved hypoxic resistance in culture and was translated into an increase in cell engraftment and functional and histological recovery induction. Cell overexpression of hemeoxygenase-1 through adenoviral transfection showed superior therapeutic capacity, mainly through protection from inflammation and apoptosis [108], whereas targeted Akt overproduction in MSC restored cardiac function 2 weeks after MI through paracrine actions, including protection from hypoxia-induced apoptosis, release of cytokines, and preservation of tissue metabolism [109–111]. Others have explored antioxidants, like Song et al. who published that reactive oxygen species (ROS) diminished BM-MSC adherence to the substrate, but when treated with an ROS scavenger (N-acetyl-L-cysteine), engraftment was improved and the increase in fibrosis and infarct size prevented [112]. Hsp20 overexpression also protected MSC from oxidative stress and improved their beneficial activities [97].

However, viral or genetic modification of cells implies certain risks that currently make it difficult for a devised therapy to reach the bedside. Bioengineering uses biocompatible materials to improve or direct cell therapy and either synthetic or naturally derived systems have been employed. Jin and coworkers seeded BM-MSC on poly(lactide-co-1-caprolactone) patches which when applied on a rat cryoinjury model were able to improve cardiac function and decrease infarct size [113]. Porcine small intestine submucosa, a decellularized substrate, has been employed to treat a rabbit model of chronic MI, showing a significant benefit upon contractility and histology, as well as cell migration towards the injured tissue [114]. The cell sheet technology allows increasing thickness through stacking of constructs, as shown by Chen et al. [115], where its transplantation in a rat syngenic model of cardiac ischemia improved cardiac function as well as paracrine secretion of therapeutic molecules by grafted cells. Dr. Mori's group compared the transplantation of a cell sheet seeded with ADSC versus fibroblasts, showing the superior effect of the mesenchymal progenitors [116]. Recently, autologous ADSC were transplanted along with allogenic ESC-derived CD15^+^ cardiac progenitors in a monkey model of infarction, demonstrating the safety of the procedure, although the functional outcome was not analyzed [117].

Finally, a word of caution must be added. Animal models of the disease are a powerful tool to explore the feasibility of a certain therapy, as MSC treatment of MI, but despite positive and reproducible results, rodent and even large animal models are just oversimplifications of the more complex setting of the human disease. As above stated, animals where cell therapy is applied are not elderly, nor severely diseased, thus making any result, even if tremendously positive, just a clue or hint before proceeding to the final application to patients, where the real safety and effectiveness can be assessed.

## 6. Mesenchymal Progenitors and Clinical Application

Several clinical trials have been performed with autologous BM-MSC, proving their safety when transplanted in patients with either acute or chronic myocardial infarction [118–120]. Moreover, the first clinical trial designed as a randomized study showed an improvement in the cardiac function 3 months after BM-MSC intracoronary infusion in patients with acute MI [120]. In view of the encouraging results of the previous clinical trials, new phase-I/II studies have been initiated, including the transendocardial autologous cells (hMSC or hBMC) in Ischemic Heart Failure Trial (TAC-HFT; http://www.clinicaltrials.org/NCT00768066/), the Prospective Randomised study Of MSC THErapy in patients Undergoing cardiac Surgery (PROMETHEUS) trial (http://www.clinicaltrials.org/NCT00587990/), and the Percutaneous Stem Cell Injection Delivery Effects on Neomyogenesis (POSEIDON) pilot study (http://www.clinicaltrials.org/NCT01087996/) [121], among others.

BM-MSCs from allogeneic origin have been tested as an off-the-shelf cell product. The first phase-I, randomized, double-blind, placebo-controlled, dose-escalation study was performed in 53 patients with acute MI, who intravenously received one of three doses of BM-MSCs (0.5, 1.6 or 5.0 × 10^6^ BM-MSC/Kg body weight) derived from a single cell donor (Prochymal; Osiris therapeutics, Inc.) or placebo [122]. Safety of the procedure was proven, showing fewer episodes of ventricular tachycardia and even a better lung function in the cell-treated group. Also, renal, hepatic, and hematologic laboratory indexes were similar in the two groups and no patient developed tumors. Importantly, a significant increase was detected in the ejection fraction (EF) of the treated patients. In a magnetic resonance imaging substudy, cell treatment, but not placebo, increased left ventricular ejection fraction and led to a reversal of adverse remodeling after 6 months of treatment. Now, a phase-II multicentre trial of ProchymalTM has been started (http://www.clinicaltrials.org/NCT00877903/).

Furthermore, BM-MSC safety has been tested in patients with moderate-to-severe chronic heart failure in a phase-II, randomized, single-blind, placebo-controlled, dose-escalation, multicenter study. In this clinical trial, the patients received an endoventricular injection of an allogeneic BM-MSC product (Revascor, Mesoblast Ltd.) along the infarct border zone and no procedure-related complications were reported. Analysis of the data obtained after 6 months of followup (http://www.mesoblast.com/newsroom/asx-announcements/archives/) showed a significant decrease in the number of patients who developed any severe or major adverse cardiac event, such as composite of cardiac death, heart attack, or need for coronary revascularization procedures. Moreover, the first cohort in the study (*n* = 20 patients), which received the low dose of the cell treatment, showed a significantly greater increase in the EF when compared with the control group [123].

On the other hand, regarding other sources of MSC such as adipose tissue, no clinical trials have been initiated yet, despite the fact that the beneficial potential of ADSC has been preclinically demonstrated [83]. Until now, only the noncultured adipose stromal vascular fraction is being tested at the clinical level. The first study, a double-blind, placebo-controlled trial named APOLLO (http://www.clinicaltrials.org/NCT00442806/; [124]) where AMI patients received autologous adipose derived stem cells by intracoronary infusion, was proven safe. Now, a phase II/III ADVANCE trial has been initiated to evaluate their efficacy (http://www.clinicaltrials.org/NCT01216995/).

In general, the results obtained from the many clinical trials performed, either with MSC or other stem cell populations (mainly BM-derived cells and skeletal myoblasts), have taught us several important lessons that will help to design and interpret the following clinical trials. (i) Cell treatment is not equally efficacious in all the patients. In general, it seems that the worse the heart damage (meaning severely decreased postrevascularization LVEF or high degree of infarct transmurality), the better the benefit induced by the transplanted cells seems to be [125–127]. (ii) Cell dose and timing for treatment are critical. Thus, a meta-analysis of the results obtained in the most relevant clinical trials performed in acute MI patients treated with BM cells has shown a significantly greater effect in those patients that received high cell doses (10^8^ cells). Also, the same study showed a greater beneficial effect when cells are infused during the first week after the infarct [128]. (iii) Autologous treatment is not necessarily the best. Until now, most of the clinical studies have been designed for autologous cell application in order to avoid the immunorejection of the transplanted cells. However, it has to be borne in mind that stem cells derived from aged patients with risk of atherosclerosis or other diseases might be defective, and thereby, treatment with them might not be as efficacious as with cells derived from young healthy donors [129–131]. In that sense, the use of MSC, which present immunomodulatory properties [132], could be of great relevance. Thus, advantages of allogeneic MSC treatment would be that, together with the putative greater paracrine effect that allogeneic cells derived from a healthy donor could exert, a fully tested clinical grade ready to use allogeneic cell product could be available for any patient. Importantly, patients with acute MI could also be eligible for such treatment. Furthermore, the logistical complexity and manufacturing costs that autologous cell preparation implies would be significantly reduced by the allogeneic application. However, caution should be taken when taking into consideration the issues related to their immune privilege explained above.

Thus, although it is mandatory to better understand the mechanisms involved in the MSC phenotype switch and to elucidate how this could affect the cells' potential benefit, it has to be considered that, in any case, because MSC would not differentiate towards cardiovascular cells and would act as a paracrine factor source [111], their permanent presence in the heart might not be necessary for therapeutic purposes. In that case, a temporarily action should be sufficient for exerting their benefit. Phase-II clinical trials are currently assessing the efficacy of the allogeneic MSC treatment, together with the long-term safety. If allogeneicity of the cells diminishes their effectiveness, several options could be considered, like temporal patient immunosuppression and/or donor-recipient HLA-II mismatch minimizing. As a consequence, the increase in the rate of engraftment of transplanted cells is so far one of the main challenges. As already indicated, the use of scaffolds could improve this factor. Interestingly, a clinical trial has been performed in 15 patients with chronic MI who were treated with a collagen scaffold previously seeded with bone marrow mononuclear cells [133]. The cellularized patch was implanted onto the pericardium and no adverse events were reported, showing the feasibility and safety of the treatment. Furthermore, a limiting effect in ventricular wall remodeling and an improved diastolic function were detected. These positive results will probably promote new larger randomized controlled trials, where mesenchymal and other stem cell populations might be tested in combination with scaffolds, thus leading to a further step in the therapeutic use of stem cells.

## 7. Conclusion

Mesenchymal cells have raised substantial interest in recent years due to their potential and versatility. Although we are only now starting to understand the mechanisms by which they repair or induce the repair of damaged organs, their pleiotropic activity and the technical ease of manipulation makes them good candidates for the treatment of the MI. Though waiting for randomized, double-blinded, placebo-controlled clinical trials in which large cohorts of patients could participate, the available data demonstrates the safety of the therapy and points towards a positive effect, further encouraging new investigations. The addition of the latest improvements in the field, including *in vitro* conditioning and bioengineering, will surely suppose a further step towards finding an optimized treatment. However, certain issues, mainly immunomodulatory capacity and allogenic use, need to be better understood.

## Figures and Tables

**Table 1:** Standardized requirements for MSC definition.

Multipotent mesenchymal stromal cells (MSE) properties
(i) Plastic adherence
(ii) Cell surface antigen expression profile
CD73^+^, CD90^+^, CD105^+^, HLA-DR^−^, CD11b^−^, CD14^−^,
CD19^−^, CD34^−^, CD45^−^, CD79*α* ^−^
(iii) Multipotency
Chondroblast, Adipocyte, Osteoblast
